# General Propagation Lattice Boltzmann Model for the Boussinesq Equation

**DOI:** 10.3390/e24040486

**Published:** 2022-03-30

**Authors:** Wei Yang, Chunguang Li

**Affiliations:** School of Mathematics and Information Science, North Minzu University, Yinchuan 750021, China; 13568357202@163.com

**Keywords:** lattice Boltzmann model, Boussinesq equation, numerical simulations, Chapman–Enskog multi-scale analysis, Taylor expansion technique

## Abstract

A general propagation lattice Boltzmann model is used to solve Boussinesq equations. Different local equilibrium distribution functions are selected, and the macroscopic equation is recovered with second order accuracy by means of the Chapman–Enskog multi-scale analysis and the Taylor expansion technique. To verify the effectiveness of the present model, some Boussinesq equations with initial boundary value problems are simulated. It is shown that our model can remain stable and accurate, which is an effective algorithm worthy of promotion and application.

## 1. Introduction

Lattice Boltzmann method (LBM) is a popular numerical method developed in recent decades [1,2,3], which is based on kinetic theory. In the lattice Boltzmann method, in addition to the fluid being discretized into fluid particles, the physical region is also discretized into a series of lattices, and the time is discretized into a series of time steps. It has unique advantages, such as easy implementation, simple boundary condition processing, and easy parallel computing [4]. It can be used to simulate some fluid flow [5,6,7] and solve partial differential equations [8,9,10,11,12].

The Boussinesq equation [13,14] describes the motions of long waves in one-dimensional nonlinear lattices and in shallow water under gravity. The numerical solutions of the Boussinesq equation are extensively studied [15,16]. Ref. [17] proposes a lattice Boltzmann model with an optimization term for the generalized Boussinesq equation. Ref. [18] simulates Boussinesq equations with source terms.

A general propagation lattice Boltzmann (GPLB) scheme is more general than the standard lattice Bhatnagar–Gross–Krook (SLBGK) model [19]. Some studies on GPLB models are being promoted [9,12,19]. The stability and accuracy of simulating equations will be improved by GPLB models. In this paper, we develop a GPLB model to solve Boussinesq equations. The effectiveness and stability of our model are verified by comparing the simulation results and the exact solutions.

An outline of our paper is given by: in Section 2, we derive a GPLB model for the generalized nonlinear Boussinesq equation. In Section 3, numerical simulations are performed. Finally, conclusions will be summarized in Section 4.

## 2. GPLB Model for Boussinesq Equations

This paper studies Boussinesq equations with the following forms,

(1) $$u_{tt}=\alpha u_{xx}+{\left(p\left(u\right)\right)}_{xx}+\beta u_{xxxx}.$$

where the macro variable u=u(x,t) represents the free movement of the fluid surface. The depth of the flowing fluid and the characteristic velocity of the corresponding long wave determined α,β, respectively. Equation (1) is a well-known generalized Boussinesq equation.

For Equation (1), the evolution law of the particle distribution function can be the corresponding discrete velocity Boltzmann equation [20] with the Bhatnagar–Gross–Krook (BGK) collision operator [1],

(2) $$\frac{\partial f_{\alpha }}{\partial t}+\xi_{\alpha }\frac{\partial f_{\alpha }}{\partial x}=-\frac{1}{\tau_{0}}\left[f_{\alpha }-f_{\alpha }^{eq}\right],$$

where $f_{\alpha }\left(x,t\right)$ is a scalar function describing the particle distribution at position *x* and time *t*, $\left\{\xi_{\alpha },\alpha =0,1,\ldots ,n-1\right\}$ is the set of discrete velocities. $f_{\alpha }^{eq}$ is the local equilibrium distribution function, and $\tau_{0}$ is the single relaxation time.

In the LBM, the DdQb model is often used to represent the dimensions and speed of the problem. Among them, *d* represents the dimension of the problem (1 represents one-dimension, 2 represents two-dimensions, 3 represents three-dimensions), *b* represents the number of lattice chains in the velocity model. In this paper, we use the D1Q5 velocity model, in which the discrete velocities can be defined as,

(3) $$\xi_{\alpha }=ce_{\alpha }=c\left\{0,1,-1,2,-2\right\}=\left\{0,c,-c,2c,-2c\right\},$$

where c=kΔx/Δt, representing the propagation speed of $f_{\alpha }$ along the lattice chain. The discrete lattice time step and space step are Δt and Δx, respectively. We use *k* to change the propagation process, which is an important value in the current method.

Equation (2) can be decomposed into collision and propagation steps for each Δt with applying the time-splitting method [19],

(4) $$\frac{\partial f_{\alpha }}{\partial t}=-\frac{1}{\tau_{0}}\left[f_{\alpha }-f_{\alpha }^{eq}\right]$$

(5) $$\frac{\partial f_{\alpha }}{\partial t}+e_{\alpha }\frac{\partial f_{\alpha }}{\partial x}=0.$$

We can choose the appropriate numerical schemes method according to the features of Equations (4) and (5).

### 2.1. GPLB Model for Boussinesq Equations

Since there is no spatial derivative term, Equation (4) is discretized into the following form with the explicit Euler scheme,

(6) $$f_{\alpha }^{{}^{\prime }}\left(x,t\right)=\left(1-\frac{1}{\tau }\right)f_{\alpha }\left(x,t\right)+\frac{1}{\tau }f_{\alpha }^{eq}\left(x,t\right).$$

We can see that there is no difference between the collision process and that in the SLBGK models.

We treat Equation (5) as follows,

(7) $$f_{\alpha }\left(x,t+\Delta t\right)=m_{0}f_{\alpha }^{{}^{\prime }}\left(x,t\right)+m_{-1}f_{\alpha }^{{}^{\prime }}\left(x-S_{i},t\right)+m_{1}f_{\alpha }^{{}^{\prime }}\left(x+S_{i},t\right),\;S_{i}=\Delta x\cdot e_{i},$$

where $m_{0},m_{-1}$ and $m_{1}$ are free parameters satisfying,

(8) $$m_{0}+m_{-1}+m_{1}=1,\;m_{-1}-m_{1}=\Delta t\cdot \frac{\xi_{\alpha }}{S_{i}}=\Delta t\frac{ce_{\alpha }}{\Delta xe_{\alpha }}=k.$$

Thus, we can get from Equation (8),

(9) $$m_{0}=1-n,\;m_{-1}=\frac{n+k}{2},\;m_{1}=\frac{n-k}{2}.$$

We introduce one parameter *n* for Equation (9). From Equation (7), we improve the propagation process. Substituting Equation (9) into Equation (7), we can obtain:

(10) $$\begin{array}{cc} f_{\alpha }\left(x,t+\Delta t\right)= & f_{\alpha }^{{}^{\prime }}\left(x,t\right)+\frac{n}{2}\left[f_{\alpha }^{{}^{\prime }}\left(x+S_{i},t\right)-2f_{\alpha }^{{}^{\prime }}\left(x,t\right)+f_{\alpha }^{{}^{\prime }}\left(x-S_{i},t\right)\right]-\frac{k}{2}\left[f_{\alpha }^{{}^{\prime }}\left(x+S_{i},t\right)\right.\\ \; & \left.-f_{\alpha }^{{}^{\prime }}\left(x-S_{i},t\right)\right]. \end{array}$$

We choose $k^{2}⩽n⩽1$ to ensure simulation stability [9].

So far, Equations (6) and (10) constitute the GPLB model for Equation (1). Next, we will recover Equation (1).

### 2.2. Recovery of Boussinesq Equations

To complete our general propagation lattice Boltzmann model for Equation (1), the multi-scale Chapman–Enskog [21] and Taylor expansions will be applied to obtain the specific expressions of the local equilibrium distribution function $f_{\alpha }^{eq}$.

Firstly, applying the Taylor expansion to $f_{\alpha }^{{}^{\prime }}\left(x+S_{i},t\right)$ and $f_{\alpha }^{{}^{\prime }}\left(x-S_{i},t\right)$, and retaining the terms up to $O(\Delta t^{5})$, we have:

(11) $$\begin{array}{cc} f_{\alpha }^{{}^{\prime }}\left(x+S_{i},t\right)= & f_{\alpha }^{{}^{\prime }}\left(x,t\right)+S_{i}\cdot \partial_{x}f_{\alpha }^{{}^{\prime }}\left(x,t\right)+\frac{{\left(S_{i}\cdot \partial_{x}\right)}^{2}}{2}f_{\alpha }^{{}^{\prime }}\left(x,t\right)+\frac{{\left(S_{i}\cdot \partial_{x}\right)}^{3}}{6}f_{\alpha }^{{}^{\prime }}\left(x,t\right)\\ \; & +\frac{{\left(S_{i}\cdot \partial_{x}\right)}^{4}}{24}f_{\alpha }^{{}^{\prime }}\left(x,t\right)+O\left(S_{i}^{5}\right)\\ \; & =f_{\alpha }^{{}^{\prime }}\left(x,t\right)+\frac{\Delta t}{k}\left(\xi_{\alpha }\cdot \partial_{x}\right)f_{\alpha }^{{}^{\prime }}\left(x,t\right)+\frac{\Delta t^{2}}{2k^{2}}{\left(\xi_{\alpha }\cdot \partial_{x}\right)}^{2}f_{\alpha }^{{}^{\prime }}\left(x,t\right)\\ \; & +\frac{\Delta t^{3}}{6k^{3}}{\left(\xi_{\alpha }\cdot \partial_{x}\right)}^{3}f_{\alpha }^{{}^{\prime }}\left(x,t\right)+\frac{\Delta t^{4}}{24k^{4}}{\left(\xi_{\alpha }\cdot \partial_{x}\right)}^{4}f_{\alpha }^{{}^{\prime }}\left(x,t\right)+O\left(\Delta t^{5}\right), \end{array}$$

(12) $$\begin{array}{cc} f_{\alpha }^{{}^{\prime }}\left(x-S_{i},t\right)= & f_{\alpha }^{{}^{\prime }}\left(x,t\right)-\frac{\Delta t}{k}\left(\xi_{\alpha }\cdot \partial_{x}\right)f_{\alpha }^{{}^{\prime }}\left(x,t\right)+\frac{\Delta t^{2}}{2k^{2}}{\left(\xi_{\alpha }\cdot \partial_{x}\right)}^{2}f_{\alpha }^{{}^{\prime }}\left(x,t\right)\\ \; & -\frac{\Delta t^{3}}{6k^{3}}{\left(\xi_{\alpha }\cdot \partial_{x}\right)}^{3}f_{\alpha }^{{}^{\prime }}\left(x,t\right)+\frac{\Delta t^{4}}{24k^{4}}{\left(\xi_{\alpha }\cdot \partial_{x}\right)}^{4}f_{\alpha }^{{}^{\prime }}\left(x,t\right)+O\left(\Delta t^{5}\right). \end{array}$$

Using Equations (10)–(12), we have:

(13) $$\begin{array}{cc} f_{\alpha }\left(x,t+\Delta t\right)= & f_{\alpha }^{{}^{\prime }}\left(x,t\right)-\Delta t\left(\xi_{\alpha }\cdot \partial_{x}\right)f_{\alpha }^{{}^{\prime }}\left(x,t\right)+\frac{\Delta t^{2}n}{2k^{2}}{\left(\xi_{\alpha }\cdot \partial_{x}\right)}^{2}f_{\alpha }^{{}^{\prime }}\left(x,t\right)\\ \; & -\frac{\Delta t^{3}}{6k^{3}}{\left(\xi_{\alpha }\cdot \partial_{x}\right)}^{3}f_{\alpha }^{{}^{\prime }}\left(x,t\right)+\frac{\Delta t^{4}n}{24k^{4}}{\left(\xi_{\alpha }\cdot \partial_{x}\right)}^{4}f_{\alpha }^{{}^{\prime }}\left(x,t\right)+O\left(\Delta t^{5}\right). \end{array}$$

Taylor expansion of Equation (13) to $O(\Delta t^{5})$,

(14) $$\begin{array}{cc} f_{\alpha }\left(x,t+\Delta t\right)= & f_{\alpha }\left(x,t\right)+\Delta t\partial_{t}f_{\alpha }\left(x,t\right)+\frac{\Delta t^{2}}{2}\partial_{t}^{2}f_{\alpha }\left(x,t\right)+\frac{\Delta t^{3}}{6}\partial_{t}^{3}f_{\alpha }\left(x,t\right)\\ \; & +\frac{\Delta t^{4}}{24}\partial_{t}^{4}f_{\alpha }\left(x,t\right)+O\left(\Delta t^{5}\right), \end{array}$$

we have:

(15) $$\begin{array}{cc} \; & f_{\alpha }\left(x,t\right)+\Delta t\partial_{t}f_{\alpha }\left(x,t\right)+\frac{\Delta t^{2}}{2}\partial_{t}^{2}f_{\alpha }\left(x,t\right)+\frac{\Delta t^{3}}{6}\partial_{t}^{3}f_{\alpha }\left(x,t\right)++\frac{\Delta t^{4}}{24}\partial_{t}^{4}f_{\alpha }\left(x,t\right)+O\left(\Delta t^{5}\right)\\ \; & =f_{\alpha }^{{}^{\prime }}\left(x,t\right)-\Delta t\left(\xi_{\alpha }\cdot \partial_{x}\right)f_{\alpha }^{{}^{\prime }}\left(x,t\right)+\frac{\Delta t^{2}n}{2k^{2}}{\left(\xi_{\alpha }\cdot \partial_{x}\right)}^{2}f_{\alpha }^{{}^{\prime }}\left(x,t\right)-\frac{\Delta t^{3}}{6k^{3}}{\left(\xi_{\alpha }\cdot \partial_{x}\right)}^{3}f_{\alpha }^{{}^{\prime }}\left(x,t\right)\\ \; & +\frac{\Delta t^{4}n}{24k^{4}}{\left(\xi_{\alpha }\cdot \partial_{x}\right)}^{4}f_{\alpha }^{{}^{\prime }}\left(x,t\right)+O\left(\Delta t^{5}\right). \end{array}$$

Multi-scale Chapman–Enskog expansion of the system in the following forms,

(16) $$\partial_{x}=\varepsilon \partial_{x_{1}},$$

(17) $$\partial_{t}=\varepsilon \partial_{t_{1}}+\varepsilon^{2}\partial_{t_{2}}+\varepsilon^{3}\partial_{t_{2}}+\varepsilon^{4}\partial_{t_{4}},$$

(18) $$f_{\alpha }=\sum_{n=0}^{\infty }\epsilon^{n}f_{\alpha }^{\left(n\right)}=f_{\alpha }^{0}+\epsilon f_{\alpha }^{\left(1\right)}+\epsilon^{2}f_{\alpha }^{\left(2\right)}+\epsilon^{3}f_{\alpha }^{\left(3\right)}+\cdots .$$

Using Equations (6), (15)–(18), we obtain:

(19) $$O\left(\varepsilon^{0}\right):\;f_{\alpha }^{0}=\left(1-\frac{1}{\tau }\right)f_{\alpha }^{0}+\frac{1}{\tau }f_{\alpha }^{eq},\;i.e.,\;f_{\alpha }^{0}=f_{\alpha }^{eq},$$

(20) $$O\left(\varepsilon^{1}\right):\;f_{\alpha }^{1}+\Delta t\partial_{t_{1}}f_{\alpha }^{0}=\left(1-\frac{1}{\tau }\right)f_{\alpha }^{1}-\Delta t\left(\xi_{\alpha }\cdot \partial_{x_{1}}\right)f_{\alpha }^{0},$$

(21) $$O\left(\varepsilon^{2}\right):\;f_{\alpha }^{2}+\Delta t\partial_{t_{2}}f_{\alpha }^{0}+\Delta t\partial_{t_{1}}f_{\alpha }^{1}+\frac{\Delta t^{2}}{2}\partial_{t_{1}}^{2}f_{\alpha }^{0}=\left(1-\frac{1}{\tau }\right)f_{\alpha }^{2}+\frac{n\Delta t^{2}}{2k^{2}}{\left(\xi_{\alpha }\cdot \partial_{x_{1}}\right)}^{2}f_{\alpha }^{0},$$

(22) $$\begin{array}{cc} \; & O\left(\varepsilon^{3}\right):\;f_{\alpha }^{3}+\Delta t\partial_{t_{1}}f_{\alpha }^{2}+\Delta t\partial_{t_{2}}f_{\alpha }^{1}+\Delta t\partial_{t_{3}}f_{\alpha }^{0}+\frac{\Delta t^{2}}{2}\partial_{t_{1}}^{2}f_{\alpha }^{1}+\frac{\Delta t^{3}}{6}\partial_{t_{1}}^{3}f_{\alpha }^{0}\\ \; & =\left(1-\frac{1}{\tau }\right)f_{\alpha }^{3}+\Delta t\left(\xi_{\alpha }\cdot \partial_{x_{1}}\right)\left(1-\frac{1}{\tau }\right)f_{\alpha }^{2}-\frac{\Delta t^{3}}{6k^{3}}{\left(\xi_{\alpha }\cdot \partial_{x_{1}}\right)}^{3}f_{\alpha }^{0}, \end{array}$$

(23) $$\begin{array}{cc} \; & O\left(\varepsilon^{4}\right):\;f_{\alpha }^{4}+\Delta t\left(\partial_{t_{1}}f_{\alpha }^{3}+\partial_{t_{2}}f_{\alpha }^{2}+\partial_{t_{3}}f_{\alpha }^{1}+\partial_{t_{4}}f_{\alpha }^{0}\right)\\ \; & +\frac{\Delta t^{2}}{2}\left(\partial_{t_{1}}^{2}f_{\alpha }^{2}+\partial_{t_{2}}^{2}f_{\alpha }^{0}+2\partial_{t_{1},t_{2}}^{2}f_{\alpha }^{1}+2\partial_{t_{1},t_{3}}^{2}f_{\alpha }^{0}\right)+\frac{\Delta t^{3}}{6}\left(\partial_{t_{1}}^{3}f_{\alpha }^{1}+3\partial_{t_{2}}\partial_{t_{1}}^{2}f_{\alpha }^{0}\right)\\ \; & +\frac{\Delta t^{4}}{24}\partial_{t_{1}}^{4}f_{\alpha }^{0}\\ \; & =\left(1-\frac{1}{\tau }\right)f_{\alpha }^{4}-\Delta t\left(\xi_{\alpha }\cdot \partial_{x_{1}}\right)\left(1-\frac{1}{\tau }\right)f_{\alpha }^{3}+\frac{n\Delta t^{2}}{2k^{2}}{\left(\xi_{\alpha }\cdot \partial_{x_{1}}\right)}^{2}\left(1-\frac{1}{\tau }\right)f_{\alpha }^{2}\\ \; & +\frac{n\Delta t^{4}}{24k^{4}}{\left(\xi_{\alpha }\cdot \partial_{x_{1}}\right)}^{4}f_{\alpha }^{0}. \end{array}$$

Simplifying Equations (19)–(23), we have:

(24) $$\begin{array}{cc} \; & -\frac{f_{\alpha }^{1}}{\tau \Delta t}=\;\left[\partial_{t_{1}}+\left(\xi_{\alpha }\cdot \partial_{x_{1}}\right)\right]f_{\alpha }^{0}, \end{array}$$

(25) $$\begin{array}{cc} \; & -\frac{f_{\alpha }^{2}}{\tau \Delta t}=\;\left[\frac{\Delta t}{2}\partial_{t_{1}}^{2}+\partial_{t_{2}}-\frac{n\Delta t}{2k^{2}}{\left(\xi_{\alpha }\cdot \partial_{x_{1}}\right)}^{2}\right]f_{\alpha }^{0}+\left[\partial_{t_{1}}+\left(1-\frac{1}{\tau }\right)\left(\xi_{\alpha }\cdot \partial_{x_{1}}\right)\right]f_{\alpha }^{1}, \end{array}$$

(26) $$\begin{array}{cc} \; & -\frac{f_{\alpha }^{3}}{\tau \Delta t}=\;\left[\frac{\Delta t^{2}}{6}\partial_{t_{1}}^{3}+\Delta t\partial_{t_{1},t_{2}}^{2}+\partial_{t_{3}}+\frac{\Delta t^{2}}{6k^{2}}{\left(\xi_{\alpha }\cdot \partial_{x_{1}}\right)}^{3}\right]f_{\alpha }^{0}+\left[\frac{\Delta t}{2}\partial_{t_{1}}^{2}+\partial_{t_{2}}-\frac{n\Delta t}{2k^{2}}\left(1-\frac{1}{\tau }\right)\right.\\ \; & \left.{\left(\xi_{\alpha }\cdot \partial_{x_{1}}\right)}^{2}\right]f_{\alpha }^{1}+\left[\partial_{t_{1}}+\left(1-\frac{1}{\tau }\right)\left(\xi_{\alpha }\cdot \partial_{x_{1}}\right)\right]f_{\alpha }^{2}, \end{array}$$

(27) $$\begin{array}{cc} \; & -\frac{f_{\alpha }^{4}}{\tau \Delta t}=\;\left[\partial_{t_{4}}+\frac{\Delta t}{2}\partial_{t_{2}}^{2}+\Delta t\partial_{t_{1},t_{3}}^{2}+\frac{\Delta t^{2}}{2}\partial_{t_{2}}\partial_{t_{1}}^{2}+\frac{\Delta t^{3}}{24}\partial_{t_{1}}^{4}-\frac{n\Delta t^{3}}{24k^{4}}{\left(\xi_{\alpha }\cdot \partial_{x_{1}}\right)}^{4}\right]f_{\alpha }^{0}\\ \; & +\left[\partial_{t_{3}}+\Delta t\partial_{t_{1},t_{2}}^{2}+\frac{\Delta t^{2}}{6}\partial_{t_{1}}^{3}+\frac{\Delta t^{2}}{6k^{3}}{\left(\xi_{\alpha }\cdot \partial_{x_{1}}\right)}^{3}\left(1-\frac{1}{\tau }\right)\right]f_{\alpha }^{1}+\left[\partial_{t_{2}}+\frac{\Delta t}{2}\partial_{t_{1}}^{2}-\frac{n\Delta t}{2k^{2}}{\left(\xi_{\alpha }\cdot \partial_{x_{1}}\right)}^{2}\right.\\ \; & \left.(1-\frac{1}{\tau })\right]f_{\alpha }^{2}+\left[\partial_{t_{1}}-\Delta t\left(\xi_{\alpha }\cdot \partial_{x_{1}}\right)\left(1-\frac{1}{\tau }\right)\right]f_{\alpha }^{3}. \end{array}$$

From Equation (24), one can obtain:

(28) $$f_{\alpha }^{1}=-\tau \Delta t\left\{\left[\partial_{t_{1}}+\left(\xi_{\alpha }\cdot \partial_{x_{1}}\right)\right]f_{\alpha }^{0}\right\}.$$

Substituting Equation (28) into (25), we have:

(29) $$\begin{array}{c} f_{\alpha }^{2}=-\tau \Delta t^{2}\left\{\left[\left(\frac{1}{2}-\tau \right)\partial_{t_{1}}^{2}+\frac{1}{\Delta t}\partial_{t_{2}}+\left(1-2\tau \right)\partial_{t_{1}}\left(\xi_{\alpha }\cdot \partial_{x_{1}}\right)-\tau_{1}{\left(\xi_{\alpha }\cdot \partial_{x_{1}}\right)}^{2}\right]f_{\alpha }^{0}\right\}. \end{array}$$

Coupling Equations (26), (28) and (29), we obtain:

(30) $$\begin{array}{cc} f_{\alpha }^{3}= & \tau \Delta t^{3}\left\{\left[\left(\tau^{2}-\tau +\frac{1}{6}\right)\partial_{t_{1}}^{3}+\frac{1}{\Delta t}\left(1-2\tau \right)\partial_{t_{1},t_{2}}^{2}+\frac{1}{\Delta t^{2}}\partial_{t_{3}}\right]+\left[\left(3\tau^{2}-\frac{3\tau }{2}-1\right)\partial_{t_{1}}^{2}\right.\right.\\ \; & \left.+\frac{1}{\Delta t}\left(1-2\tau \right)\partial_{t_{2}}\right]\left(\xi_{\alpha }\cdot \partial_{x_{1}}\right)+\left[3\tau^{2}+\left(\frac{n}{k^{2}}-4\right)\tau +1-\frac{n}{2k^{2}}\right]\partial_{t_{1}}{\left(\xi_{\alpha }\cdot \partial_{x_{1}}\right)}^{2}\\ \; & \left.+\left[\left(\frac{n}{k^{2}}-1\right)\tau +1-\frac{n}{k^{2}}+\frac{1}{6k^{2}}\right]{\left(\xi_{\alpha }\cdot \partial_{x_{1}}\right)}^{3}\right\}f_{\alpha }^{0}. \end{array}$$

Lastly, substituting Equations (28)–(30) into Equation (27), we have:

(31) $$\begin{array}{cc} \;f_{\alpha }^{4}= & \tau \Delta t^{4}\left\{\left[\frac{1}{\Delta t^{3}}\partial_{t_{4}}+\left(\frac{1}{2\Delta t^{2}}-\frac{\tau }{\Delta t^{2}}\right)\partial_{t_{2}}^{2}+\left(\frac{1}{\Delta t^{2}}-\frac{2\tau }{\Delta t^{2}}\right)\partial_{t_{1},t_{3}}^{2}+\left(\frac{1}{2\Delta t}\right.\right.\right.\\ \; & \left.\left.\left.-\frac{3\tau }{\Delta t}+\frac{3\tau^{2}}{\Delta t}\right)\partial_{t_{2}}\partial_{t_{1}}^{2}+\tau_{2}\partial_{t_{1}}^{4}\right]+\left[\left(-\left(\frac{1}{\Delta t}-\frac{\tau }{\Delta t^{2}}\right)\tau +\frac{1}{\Delta t}\right)\partial_{t_{3}}\right.\right.\\ \; & \left.\left.+\tau_{3}\partial_{t_{1},t_{2}}^{2}+\tau_{4}\partial_{t_{1}}^{3}\right]\left(\xi_{\alpha }\cdot \partial_{x_{1}}\right)+\tau_{5}{\left(\xi_{\alpha }\cdot \partial_{x_{1}}\right)}^{4}+\tau_{6}\partial_{t_{1}}^{2}{\left(\xi_{\alpha }\cdot \partial_{x_{1}}\right)}^{3}\right.\\ \; & \left.+\left(\tau_{7}\partial_{t_{1}}^{2}+\tau_{8}\partial_{t_{2}}\right){\left(\xi_{\alpha }\cdot \partial_{x_{1}}\right)}^{2}\right\}f_{\alpha }^{0}, \end{array}$$

where:

$$\begin{array}{cc} \;\; & \tau_{1}=\tau -1+\frac{n}{2k^{2}},\\ \; & \tau_{2}=-\tau^{3}+\frac{3}{2}\tau^{2}-\left(\frac{\Delta t}{6}+\frac{5}{12}\right)\tau +\frac{1}{24},\\ \; & \tau_{3}=\left(2+\frac{4}{\Delta t}\right)\tau^{2}-\left(\frac{3}{\Delta t}+3\right)\tau +1,\\ \; & \tau_{4}=-\tau^{3}\Delta t+2\tau^{2}\Delta t-\frac{7\Delta t}{6}\tau +\frac{\Delta t}{6},\\ \; & \tau_{5}=\left(-\frac{n}{2k^{2}}-\frac{n\Delta t}{k^{2}}+\Delta t\right)\tau^{2}+\left(-\frac{1}{6k^{3}}+\frac{n}{k^{2}}-\frac{n^{2}}{4k^{4}}+\frac{2n\Delta t}{k^{2}}-2\Delta t-\frac{\Delta t}{6k^{2}}\right)\tau -\frac{n}{24k^{4}}+\frac{1}{6k^{3}}\\ \; & -\frac{n}{2k^{2}}+\frac{n^{2}}{4k^{4}}-\frac{n\Delta t}{k^{2}}+\frac{\Delta t}{6k^{2}}+\Delta t,\\ \; & \tau_{6}=-3\Delta t\tau^{3}+\left(-\frac{2n}{k^{2}}+7\Delta t-\frac{n\Delta t}{k^{2}}+1\right)\tau^{2}+\left(\frac{5n}{2k^{2}}-\frac{2}{6k^{3}}+\frac{3n\Delta t}{k^{2}}-\Delta t-1\right)+\frac{1}{6k^{3}}-\frac{n}{2k^{2}}\\ \; & -3\Delta t-\frac{n\Delta t}{2k^{2}},\\ \; & \tau_{7}=\left(\frac{9}{2}-\frac{3n}{2k^{2}}-\frac{9\Delta t}{2}\right)\tau^{2}+\left(3\Delta t-3\right)\tau^{3}+\left(\frac{3n}{2k^{2}}+\frac{\Delta t}{2}-\frac{3}{2}\right)\tau +\Delta t-\frac{n}{4k^{2}},\\ \; & \tau_{8}=\left(2+\frac{1}{\Delta t}\right)\tau^{2}+\left(-3+\frac{1}{\Delta t}+\frac{n}{k^{2}\Delta t}\right)\tau +1-\frac{n}{2k^{2}\Delta t}. \end{array}$$

$f_{\alpha }$ and $f_{\alpha }^{eq}$ follow:

(32) $$\sum_{\alpha }f_{\alpha }=\sum_{\alpha }f_{\alpha }^{eq}=u_{t}.$$

From Equation (19), we have:

(33) $$\sum_{\alpha }f_{\alpha }^{0}=u_{t},\;\sum_{\alpha }f_{\alpha }^{n}=0,\;n>0.$$

In order to recover Equation (1) and satisfy the solvability, based on ensuring that the truncation error is minimized, it is determined that $f_{\alpha }$ and $f_{\alpha }^{eq}$ should satisfy the following moment conditions:

(34) $$\begin{array}{c} \sum_{\alpha }\xi_{\alpha }f_{\alpha }^{0}=0, \end{array}$$

(35) $$\begin{array}{c} \;\;\;\;\sum_{\alpha }\xi_{\alpha }^{2}f_{\alpha }^{0}=\frac{\alpha u+p(u)}{\tau_{1}\Delta t}, \end{array}$$

(36) $$\begin{array}{c} \sum_{\alpha }\xi_{\alpha }^{3}f_{\alpha }^{0}=0, \end{array}$$

(37) $$\begin{array}{c} \;\;\;\sum_{\alpha }\xi_{\alpha }^{4}f_{\alpha }^{0}=-\frac{\beta u}{\tau_{5}\Delta t^{3}}. \end{array}$$

Summing Equation (28) over α, and coupling with Equations (32) and (33), we obtain:

(38) $$\begin{array}{c} \partial_{t_{1}}\left(u_{t}\right)\;=0. \end{array}$$

Summing Equation (29) over α and coupling with Equations (33)–(35), we obtain:

(39) $$\partial_{t_{2}}\left(u_{t}\right)\;=\frac{1}{\varepsilon^{2}}\left(\alpha u_{xx}+{\left(p(u)\right)}_{xx}\right).$$

Summing Equation (30) over α and coupling with Equations (33)–(36), we obtain:

(40) $$\partial_{t_{3}}\left(u_{t}\right)\;=-\frac{\Delta t^{2}}{\varepsilon^{2}}\left[3\tau^{2}+\left(\frac{n}{k^{2}}-4\right)\tau +1-\frac{n}{2k^{2}}\right]\partial_{t_{1}}\left[\frac{\alpha u_{xx}+{\left(p(u)\right)}_{xx}}{\Delta t\tau_{1}}\right].$$

Summing Equation (31) over α and using Equations (33)–(37), we obtain:

(41) $$\partial_{t_{4}}\left(u_{t}\right)\;=-\frac{\Delta t^{3}}{\varepsilon^{2}}\left(\frac{1}{2\Delta t^{2}}-\frac{\tau }{\Delta t^{2}}\right)\left(\alpha u_{xx}+{\left(p(u)\right)}_{xx}\right)+\frac{1}{\varepsilon^{4}}\partial_{x}^{4}\left(\beta u\right)-\frac{\Delta t^{3}}{\varepsilon^{2}}\tau_{8}\partial_{t_{2}}\left[\frac{\alpha u_{xx}+{\left(p(u)\right)}_{xx}}{\Delta t\tau_{1}}\right].$$

Taking Equation (38) × ε+ Equation (39) × $\varepsilon^{2}+$ Equation (40) × $\varepsilon^{3}+$ Equation (41) × $\varepsilon^{4}$ and assuming ε=Δt, macroscopic equations, i.e., Equation (1), can be recovered with second order accuracy,

(42) $$u_{tt}-\alpha u_{xx}-{\left(p\left(u\right)\right)}_{xx}-\beta u_{xxxx}=R.$$

Through error analysis, we have:

$$\begin{array}{cc} R= & -\frac{\varepsilon^{2}}{\tau_{1}}\left[3\tau^{2}+\left(\frac{n}{k^{2}}-4\right)\tau +1-\frac{n}{2k^{2}}\right]\left\{\alpha \partial_{t_{1}}\left(u_{xx}\right)+\partial_{t_{1}}\left[{\left(p(u)\right)}_{xx}\right]\right\}-\varepsilon^{3}\left(\frac{1}{2}-\tau \right)\\ \; & \left\{\alpha \partial_{t_{2}}\left(u_{xx}\right)+\partial_{t_{2}}\left[{\left(p(u)\right)}_{xx}\right]\right\}-\frac{\varepsilon^{3}}{\tau_{1}}\left[\left(2\varepsilon +1\right)\tau^{2}+\left(-3\varepsilon +1+\frac{n}{k^{2}}\varepsilon \right)\tau +\varepsilon -\frac{n}{2k^{2}}\right]\\ \; & \left\{\alpha \partial_{t_{2}}\left(u_{xx}\right)+\partial_{t_{2}}\left[{\left(p(u)\right)}_{xx}\right]\right\}\\ \; & =O(\varepsilon^{2}). \end{array}$$

### 2.3. Equilibrium Distribution Functions

In this paper, the D1Q5 velocity model is used, i.e., b=5. The discrete velocity set is $\xi_{\alpha }=\left\{0,c,-c,2c,-2c\right\}$. Coupling with Equations (34)–(37), we can derive the equilibrium distribution functions,

(43) $$\begin{array}{c} \;\;f_{0}^{0}=u_{t}-\frac{5}{4}\left(\frac{\alpha u+p(u)}{c^{2}\Delta t\tau_{1}}\right)+\frac{1}{4}\left(\frac{\beta u}{c^{4}\Delta t\tau_{5}}\right), \end{array}$$

(44) $$\begin{array}{c} f_{1}^{0}=\frac{2}{3}\left(\frac{\alpha u+p(u)}{c^{2}\Delta t\tau_{1}}\right)-\frac{1}{6}\left(\frac{\beta u}{c^{4}\Delta t^{3}\tau_{5}}\right), \end{array}$$

(45) $$\begin{array}{c} f_{2}^{0}=\frac{2}{3}\left(\frac{\alpha u+p(u)}{c^{2}\Delta t\tau_{1}}\right)-\frac{1}{6}\left(\frac{\beta u}{c^{4}\Delta t^{3}\tau_{5}}\right), \end{array}$$

(46) $$\begin{array}{c} f_{3}^{0}=-\frac{1}{24}\left(\frac{\alpha u+p(u)}{c^{2}\Delta t\tau_{1}}-\frac{\beta u}{c^{4}\Delta t^{3}\tau_{5}}\right), \end{array}$$

(47) $$\begin{array}{c} \;\;f_{4}^{0}=-\frac{1}{24}\left(\frac{\alpha u+p(u)}{c^{2}\Delta t\tau_{1}}-\frac{\beta u}{c^{4}\Delta t^{3}\tau_{5}}\right),\; \end{array}$$

where $f_{1}^{0}=f_{2}^{0},f_{3}^{0}=f_{4}^{0}$.

## 3. Numerical Simulations

Different values of *k* and *n* determine the relationship between the GPLB and SLBGK models:

(I) k=n=1, the SLBGK scheme;
(II) $n=k^{2}$, the LW scheme;
(III) $k^{2}<n<k$, here we choose $n=\left(k+k^{2}\right)/2$;
(IV) n=k, the FP scheme;
(V) k<n<1, here, we choose n=k+0.1.

Among them, the LW scheme (II) performs better than other schemes.

To illustrate the GPLB model constructed by the combination of Equations (6) and (10), the numerical simulations of Equation (1) are developed. With taking the LW scheme, the non-equilibrium extrapolation scheme is used to treat the boundary condition [22]. In each of the test examples, the exact solution determines the initial and boundary condition. We use $E_{2}$ (root-mean-square error), $E_{\infty }$(maximum absolute error) and GRE (global relative error) to measure accuracy of our model by comparing the LBM solutions with the exact solutions [23].

(48) $$\begin{array}{c} \;\;\;\;E_{2}=\frac{1}{M}\sqrt{\sum_{i=1}^{M}{\left[u\left(x_{i},t\right)-u^{{}^{\prime }}\left(x_{i},t\right)\right]}^{2}}, \end{array}$$

(49) $$\begin{array}{c} \;\;\;E_{\infty }=\max_{i=1,2,\ldots ,M}\left|u\left(x_{i},t\right)-u^{{}^{\prime }}\left(x_{i},t\right)\right|, \end{array}$$

(50) $$\begin{array}{c} \;\;\;GRE=\frac{\sum_{i=1}^{M}\left|u\left(x_{i},t\right)-u^{{}^{\prime }}\left(x_{i},t\right)\right|}{\sum_{i=1}^{M}\left|u^{{}^{\prime }}\left(x_{i},t\right)\right|}.\; \end{array}$$

In our model, $u(x_{i},t)$ represents the LBM solution, $u^{\prime }\left(x_{i},t\right)$ represents the exact solution.The number of lattices is *M*.

**Example** **1.** *When α=0,β=−1, and $p\left(u\right)=u^{2}$, and Equation *(1)* is as follows,*

(51) $$u_{tt}-{\left(u^{2}\right)}_{xx}+u_{xxxx}=0.$$

Ref. [24] gives the exact solution u(x,t) of Equation (51)

(52) $$u\left(x,t\right)=\frac{1}{2}b^{2}-4s^{2}+6s^{2}\tanh^{2}\left[s\left(x-bt\right)\right],$$

where *b* and *s* are arbitrary constants.

**Figure 1:**
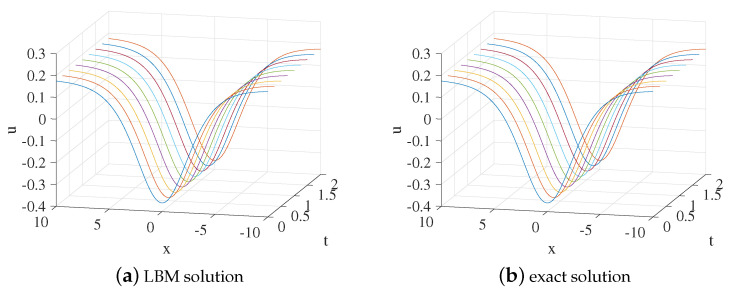
LBM solution (**a**) and exact solution (**b**) for the propagation of the soliton from t=0 to t=2 for Example 1.

**Figure 2:**
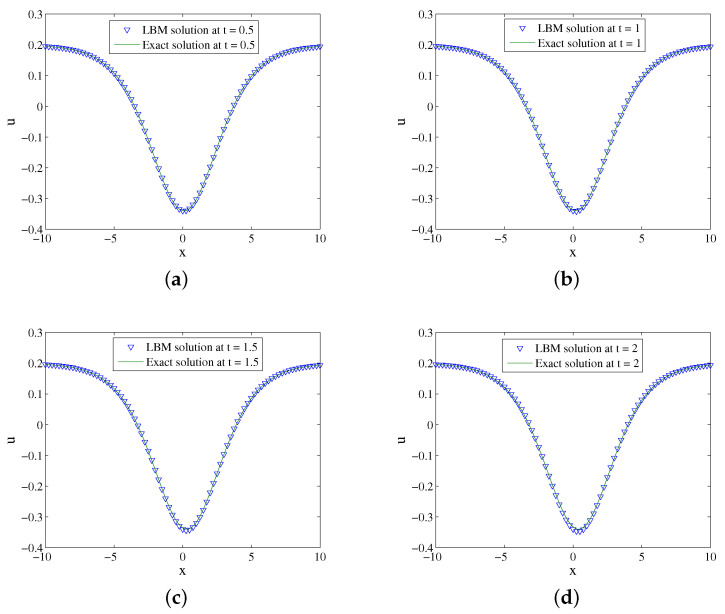
LBM solutions versus exact solutions at (**a**) t=0.5; (**b**) t=1; (**c**) t=1.5; (**d**) t=2 for Example 1.

We set x∈[−10,10], $b=0.2,s=0.3,k=0.9,n=k^{2},\tau =4,\Delta x=0.025$, and Δt=0.00025. Figure 1 shows the evolution process of numerical solution and exact solution with time. It can be seen from Figure 1 that the soliton propagates along the negative direction of the *x*-axis. The space-time evolution graph of the LBM solution and exact solution are listed in Figure 2. Table 1 lists the error of the LBM solutions at different times. The LBM solutions agree with the exact solutions well.

**Example** **2.** *When α=1,β=1, and $p\left(u\right)=3u^{2}$, and Equation *(1)* is as follows,*

(53) $$u_{tt}-u_{xx}-3{\left(u^{2}\right)}_{xx}-u_{xxxx}=0.$$

Ref. [25] gives the exact solution u(x,t) of Equation (53).

(54) $$u\left(x,t\right)=2\frac{ar^{2}\exp\left(rx+r\sqrt{1+r^{2}}t\right)}{{\left(1+a\exp\left(rx+r\sqrt{1+r^{2}}t\right)\right)}^{2}}.$$

**Figure 3:**
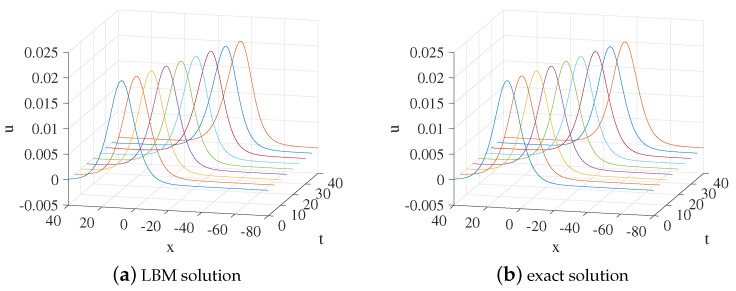
LBM solution (**a**) and exact solution (**b**) for the propagation of the solution from t=0 to t=40 for Example 2.

**Figure 4:**
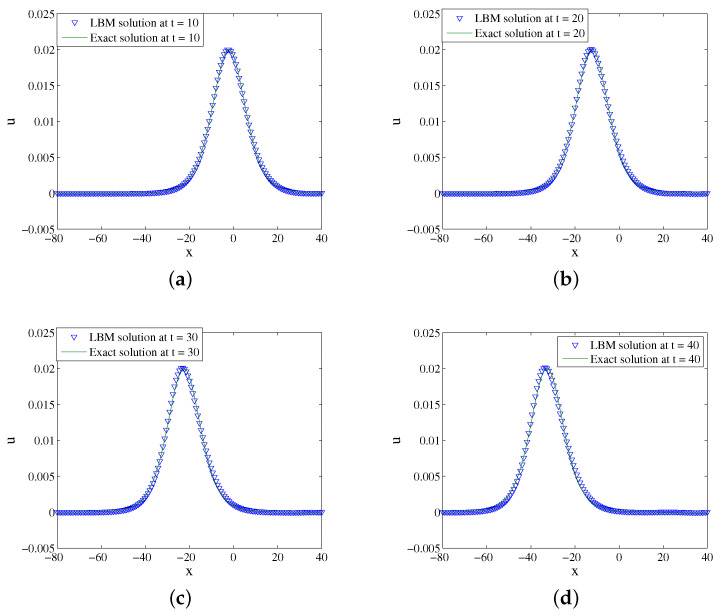
LBM solutions versus exact solutions at (**a**) t=10; (**b**) t=20; (**c**) t=30; (**d**) t=40 for Example 2.

We set x∈[−80,40], $a=0.2,r=0.2,k=0.5,n=k^{2},\tau =3.9,\Delta x=0.25$, and Δt=0.00025. Figure 3 shows the evolution process of the numerical solution and exact solution with time. It can be seen from Figure 3 that the solution propagates along the negative direction of the *x*-axis. The space-time evolution graph of the LBM solution and exact solution is shown in Figure 4. Table 2 lists the error of the LBM solutions at different times. The LBM solutions agree with the exact solutions well.

**Example** **3.** *We set α=1,β=−1, and $p\left(u\right)=u^{2}$, Equation *(1)* becomes the good nonlinear Boussinesq equation, which is of the following form,*

(55) $$u_{tt}=u_{xx}+{\left(u^{2}\right)}_{xx}-u_{xxxx}.$$

The exact solution u(x,t) of Equation (55) is as follows,


(56) $$u\left(x,t\right)=-Asech^{2}\left(\sqrt{\frac{A}{6}}\left(x+\sqrt{1-\frac{2A}{3}}t+x_{0}\right)\right).$$

**Figure 5:**
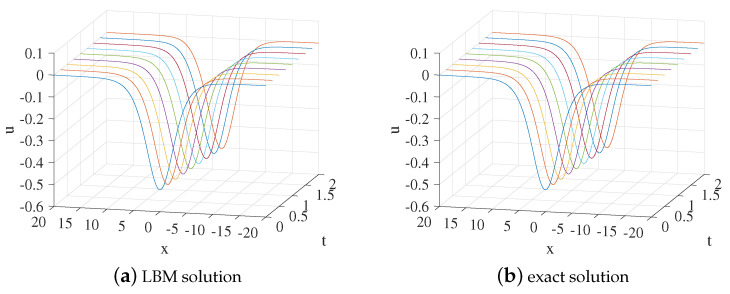
LBM solution (**a**) and exact solution (**b**) for the propagation of the solution from t=0 to t=2 for Example 3.

**Figure 6:**
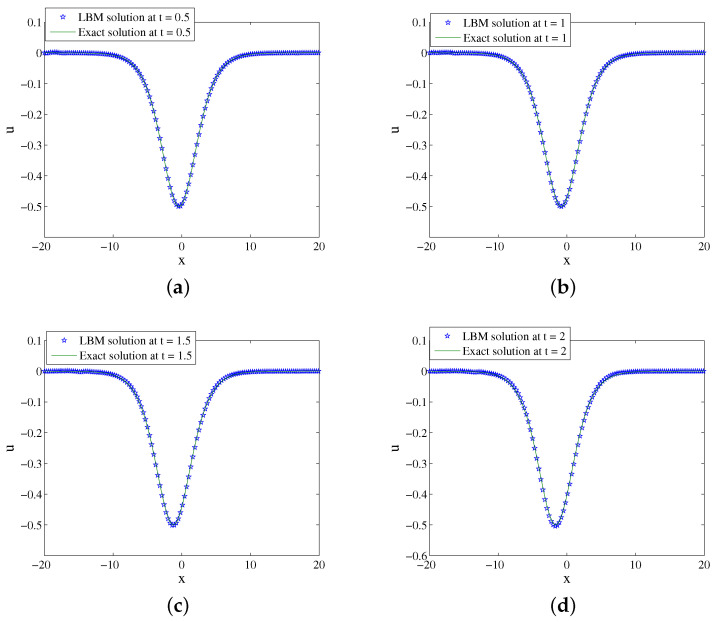
LBM solutions versus exact solutions at (**a**) t = 0.5; (**b**) t = 1; (**c**) t = 1.5; (**d**) t = 2 for Example 3.

In the computational domain of simulation, x∈[−20,20]. $A=0.5,\;x_{0}=0,$τ=1.8,Δx=0.1, and Δt=0.0001 are setted. Figure 5 shows the evolution process of the numerical solution and exact solution with time. It can be seen from Figure 5 that the solution propagates along the negative direction of the *x*-axis. The space-time evolution graph of the LBM solution and exact solution are shown in Figure 6. Table 3 lists the error of the LBM solutions at different times. The LBM solutions agree with the exact solutions well.

**Example** **4.** *We set α=1,β=1, and $p\left(u\right)=u^{2}$, Equation *(1)* becomes the bad Boussinesq equation, which is of the following form,*

(57) $$u_{tt}=u_{xx}+{\left(u^{2}\right)}_{xx}+u_{xxxx}.$$

The exact solution u(x,t) of Equation (57) is as follows,

(58) $$u\left(x,t\right)=Asech^{2}\left(\sqrt{\frac{A}{6}}\left(x+\sqrt{1+\frac{2A}{3}}t+x_{0}\right)\right).$$

**Figure 7:**
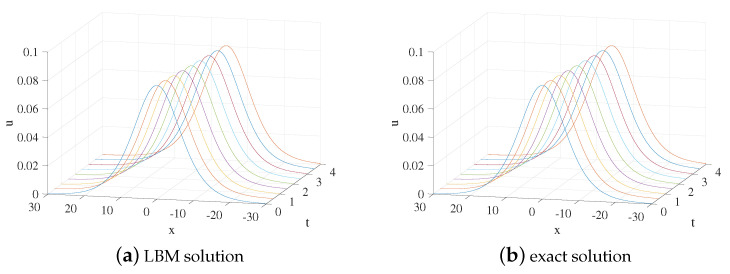
LBM solution (**a**) and exact solution (**b**) for the propagation of the solution from t=0 to t=4 for Example 4.

**Figure 8:**
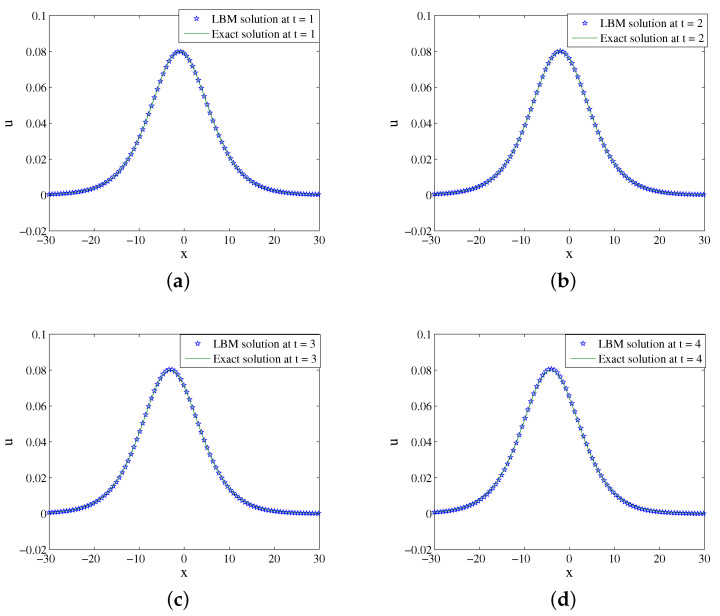
LBM solutions versus exact solutions at (**a**) t=1; (**b**) t=2; (**c**) t=3; (**d**) t=4 for Example 4.

In the computational domain of the simulation, x∈[−30,30]. $A=0.08,x_{0}=0,\tau =16,\Delta x=0.065$, and Δt=0.0001 are setted. Figure 7 shows the evolution process of numerical solution and exact solution with time. It can be seen from Figure 7 that the solution propagates along the negative direction of the *x*-axis. The space-time evolution graph of the LBM solution and exact solution are shown in Figure 8. Table 4 lists the error of the LBM solutions at different times. The LBM solutions agree with the exact solutions well.

## 4. Conclusions

In this paper, we have developed a general propagation lattice Boltzmann model for the generalized nonlinear Boussinesq equation. The macroscopic equation is recovered correctly from our model with the second-order accuracy through the Chapman–Enskog analysis. By applying the D1Q5 velocity model, Boussinesq equations are simulated. The numerical results agree well with the exact solutions with selecting the appropriate parameters that affect the propagation process. The results show that our model can remain stable and accurate, which is an effective algorithm worthy of promotion and application.

## Figures and Tables

**Table 1:** Comparison of LBM solutions and exact solutions for Example 1 at different times.

	t=0.5	t=1	t=1.5	t=2
$E_{2}$	$1.7818\times 10^{-4}$	$7.1242\times 10^{-4}$	$1.6018\times 10^{-3}$	$2.8446\times 10^{-3}$
$E_{\infty }$	$5.0682\times 10^{-4}$	$2.0260\times 10^{-3}$	$4.5535\times 10^{-3}$	$8.0828\times 10^{-3}$
GRE	$7.7264\times 10^{-4}$	$3.0896\times 10^{-3}$	$6.9474\times 10^{-3}$	$1.2341\times 10^{-2}$

**Table 2:** Comparison of LBM solutions and exact solutions for Example 2 at different times.

	t=10	t=20	t=30	t=40
$E_{2}$	$3.0848\times 10^{-6}$	$6.1918\times 10^{-6}$	$9.2945\times 10^{-6}$	$1.2367\times 10^{-5}$
$E_{\infty }$	$2.2720\times 10^{-4}$	$4.3032e\times 10^{-4}$	$6.2525\times 10^{-4}$	$8.2072\times 10^{-4}$
GRE	$1.2330\times 10^{-2}$	$2.5070\times 10^{-2}$	$3.7928\times 10^{-2}$	$5.1775\times 10^{-2}$

**Table 3:** Comparison of LBM solutions and exact solutions for Example 3 at different times.

	t=0.5	t=1	t=1.5	t=2
$E_{2}$	$1.3459\times 10^{-5}$	$4.9778\times 10^{-5}$	$1.0154\times 10^{-4}$	$1.6501\times 10^{-4}$
$E_{\infty }$	$2.1978\times 10^{-3}$	$2.8161\times 10^{-3}$	$5.3003\times 10^{-3}$	$7.5997\times 10^{-3}$
GRE	$2.6839\times 10^{-3}$	$8.2844\times 10^{-3}$	$1.6677\times 10^{-2}$	$2.7141\times 10^{-2}$

**Table 4:** Comparison of LBM solutions and exact solutions for Example 4 at different times.

	t=1	t=2	t=3	t=4
$E_{2}$	$8.4609\times 10^{-7}$	$3.2913\times 10^{-6}$	$7.2237\times 10^{-6}$	$1.2485\times 10^{-5}$
$E_{\infty }$	$4.8385\times 10^{-4}$	$1.8602\times 10^{-4}$	$4.0126\times 10^{-4}$	$6.9719\times 10^{-4}$
GRE	$9.3621\times 10^{-4}$	$3.6597\times 10^{-3}$	$8.0689\times 10^{-3}$	$1.4017\times 10^{-2}$

## Data Availability

Not applicable.
